# Biosimilars in Developed and Developing East and Southeast Asian Countries: Japan, South Korea, and Malaysia—Overview, Evolution, and Regulations Assessment

**DOI:** 10.1155/2016/5910403

**Published:** 2016-04-24

**Authors:** Tomas Gabriel Bas, Carolina Oliu Castillo

**Affiliations:** Institute of Innovation Based on Science, University of Talca, Avenida Lircay S/N, 3460000 Talca, Chile

## Abstract

The development of biological products has experienced continuous growth over the past three decades. The expiration of patent protection for many biological medicines has led to the development of biosimilars in many countries around the world. This paper reviews the literature on biosimilar drugs and covers their therapeutic status, clinical trials, approved biosimilars, and regulatory guidelines in Japan, South Korea, and Malaysia. The literature suggests that biosimilars are comparable but not identical to the reference product. They are not a generic version of an innovative product and do not ensure therapeutic equivalence. Biosimilars present more challenges than conventional generics and their marketing approval is also much more complicated. Guidelines for biosimilars were published in Japan in July 2009 by the Ministry of Health, Labour and Welfare (MHLW), in South Korea in March 2009 by the Ministry of Food and Drug Safety (MFDS), and in Malaysia in July 2008 by the National Pharmaceutical Control Bureau (NPCB).

## 1. Introduction

**Figure 1:**
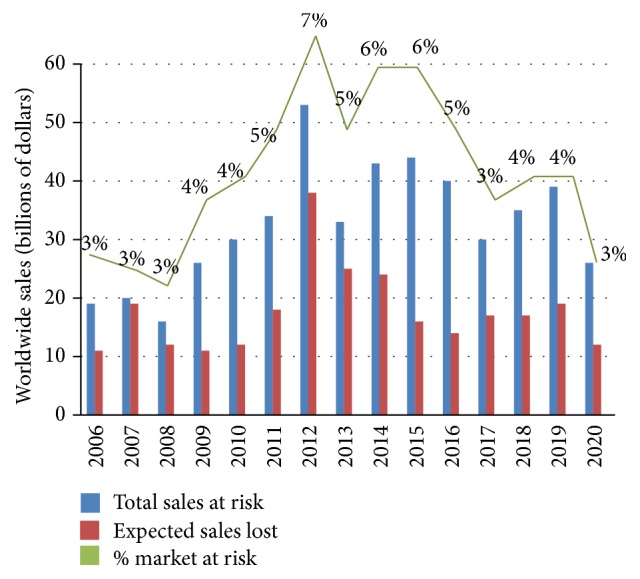
Global sales at risk due to loss of patents (2006–2020). Source: EvaluatePharma, 2014 [2].

Since its beginnings, the biopharmaceutical industry (which manufactures products of biological origin) has been constantly evolving in order to meet the new therapeutic needs of society. This evolution has generally been driven by strong investment in research and development, which has allowed it to be recognized as a continuously growing industry, based on innovative products with high margins and returns. However, this vision is far from representing the current situation of the industry, which is subject to strong government pressure and erosion of prices of their products, as a result of the growth of the generic drug industry and its immersion in a highly competitive environment in all therapeutic areas, with no single company having a dominant role in the market [1]. This situation has become worse due to the massive loss of exclusivity of patents and is further deteriorated as time passes. In 2012, 7% of worldwide sales (US $53 billion) were at risk as a result of the free entry of generic drugs, which compete directly with the original brands, forcing them to lower their prices or, even worse, to stop producing them. By 2020, it is expected that this loss of profitability will reach US $259 billion, although the predicted erosion after expiration is expected to be softer, as Figure 1 shows.

Of these products, more than 55% are products of biological and not chemical origin, opening up a great opportunity for new players in the development of biosimilars, with lower production costs and shorter development time (five to eight years) than an original medicine because of not having to follow the protocols of an unprecedented biological molecule. It is expected that biosimilars will be created in a fast-growing area of the biopharmaceutical industry and that they will become a viable alternative to compete with generic products.

To better understand the earliest references to the biopharmaceutical industry, it is very important to define the different industries that might be involved in one way or another in the development of biosimilars: the pharmaceutical industry, the biopharmaceutical industry, and, of course, the biosimilar industry. The terms usually used in science and industry concerning these industries or sectors are mixed depending on the country or region. However, we can globally define the pharmaceutical industry as one that is related to the research and process development and production, as well as marketing and/or distribution, of pharmaceuticals of chemical origin (small molecules) for therapeutic, prophylactic, and diagnostic purposes. The term referring to the biopharmaceutical industry involves the research and process development and production, including their marketing and/or distribution, of drugs of biological origin (large molecules) for therapeutic, prophylactic, and diagnostic uses [3]. Finally, the term biosimilar can be defined following the Biologics Price Competition and Innovation Act (BPCIA) as “*a compound with a high degree of similarity to their biological compound pioneer or innovator*,* but with the possibility of presenting small differences in components of clinically inactive molecule*.” However, a biosimilar product may not differ significantly in terms of the degree of purity, potency, and safety of the product being administered to the patient [4].

Large multinational firms and the Chief Research Officers (CROs) of their partners are already developing the business of biopharmaceuticals as an alternative way for obtaining profitability. Thus, from the already tested biological molecules (known as blockbusters), whose patents have expired or are on the verge of doing so, they are giving a “second life” to such molecules, including some with new therapeutic applications, but, as Table 1 shows, at a more affordable cost ranging from US $10 million to US $250 million and with clinical trials lasting between six and nine years, bringing about the emergence of “biosimilars” [5].

The United States, Europe, and Japan are the regions that invest the most in research and development of biological products. Therefore, they are expected to become the most attractive markets for the development of biosimilars. Sackman and Kuchenreuther [6] estimate that the global biosimilars market earned revenues of about US $1.3 billion in 2013. The main biological products that have attracted the development of biosimilars are Avastin, Enbrel, Herceptin, Humira, and Rituxan, which together generate more than US $50 billion annually. The costs involved in developing a biosimilar range from US $100 to US $200 million, in contrast to that of the original biological drug whose cost varies from US $800 million to US $1.3 billion. This difference may allow a price reduction between 10% and 35% in comparison to the original molecule. In Europe, where 19 biosimilars are authorized and competing with the biological product, they are sold at a price that is 30% lower than that of the original drug. With the approval of the first biosimilar in the United States in March 2015 (Filgrastim biosimilar by Sandoz) and the ongoing patent expiration of 12 of the most important biological products sold in that country, it is expected for the biosimilars market to competitively penetrate biological products in the coming years. The main actors trying to enter the biosimilars market in the United States are Hospira, Mylan, Amgen, Pfizer, and Sandoz [6].

While the biosimilars market has been dominated at this early stage by Europe and the United States, it is slowly emerging in countries with already installed biopharmaceutical infrastructure and companies. However, it is essential to consider some aspects in these countries, such as regulations, guidelines for products that fit the local market, development of therapeutic areas, economic margins that can be obtained from manufacturing (since they are less costly, they also leave a smaller margin than the original products), access to the necessary infrastructure, and potential strategic alliances. By addressing these issues, hopefully in the near future, the development of biosimilars can be converted into an appropriate economic model for countries that are emerging in these technologies. In this context, this paper presents an overview, evolution, and regulations assessment, while it also discusses the beginnings and development of biosimilars in Japan, South Korea, and Malaysia.

## 2. Materials and Methods

In order to assess the current status of biosimilars in the countries of interest, an information search strategy was designed, based on secondary sources such as government documents, official statistics, technical reports, review articles, and international journals, without language restriction that allowed establishing a regulatory framework and a diagnosis of the development of biosimilars. The keywords used in the search to process information include* biosimilars, development of biosimilars, biopharmaceutical companies, non-patent biologics, clinical trial, regulatory biosimilars, follow-on biologics, generic, molecule, biomedicine, data-marketing exclusivity, evolution, developing trials, and therapeutic.*

Information search was also conducted through the brand names and active components of each currently marketed biosimilar. A search of the websites of World Health Organization (WHO), the Food and Drug Administration of the United States (FDA), the European Medicines Agency (EMA), the Ministry of Health, Labour and Welfare (MHLW) of Japan, the Ministry of Food and Drug Safety (MFDS) of South Korea, and the National Pharmaceutical Control Bureau (NPCB) of Malaysia and overall regulatory authorities also was conducted for legislative decisions, guidance, and evaluation for approved biosimilars. A search for information on the main companies developing biosimilars in the countries of interest was also performed, allowing us to have an appreciation of the history of the biopharmaceutical industry and the changes it had undergone for the development of these new drugs.

## 3. **Results and Discussion**

### 3.1. Japan

#### 3.1.1. Pharmaceutical and Biopharmaceutical Industries in Japan

The Japanese pharmaceutical market is the second most important one in the world after the United States, with sales of over US $143 billion in 2014 [8], representing 14% of the global pharmaceutical market. Despite this, the sector has been forced to reduce production costs and increase its speed to market products in order to continue obtaining profitability as a result of the recent Asian economic crisis. This situation caused an expected reduction in investment rates in R&D by the private sector and a decline in the growth rate of the country. Therefore, the government has had to develop strategies to revitalize the industrial sector. This has been achieved through the creation of tax incentives for companies that make further innovations in their products (e.g., biosimilars) above that of generic products or others showing low level of innovation [9]. These measures have led the country to enhance local research and development.

The biopharmaceutical market in Japan is of great importance to the world. However, most of the products sold there are of foreign origin but manufactured locally under license. Moreover, as Table 2 shows, local companies are tripping over themselves to carry out research and development, with a marked delay in comparison to what is happening with drugs of chemical origin.

Of the nine most important biological drugs sold in Japan (according to level of sales), only one (epoetin alfa BS) is of Japanese origin, which demonstrates the low local impact of research and development on these types of products.

#### 3.1.2. Regulatory Guidelines for Biosimilars in Japan

The Ministry of Health, Labour and Welfare (MHLW) is the regulatory body in Japan, responsible for the scientific evaluation of medicines developed by pharmaceutical companies to be used in Japan and for making decisions on approval of drugs, including biologicals. It consists of three main areas [10]:

- *Standards for Quality Assurance of Drugs, Quasidrugs, Cosmetics, and Medical Devices*. They are up to ministerial standards on good clinical practice and quality assurance of drugs.
- *Regulations for Buildings and Facilities for Pharmacies and So On*. Regulations are established on the monitoring of the physical facilities that manufacture and distribute drugs.
- *Standards for Manufacturing Control and Quality Control of Drugs and Quasidrugs*. They regulate the field of good manufacturing practices.

There is also the Pharmaceuticals and Medical Devices Agency (PMDA), working together with the MHLW. The PMDA conducts scientific reviews of marketing authorization applications of pharmaceuticals and medical devices and monitoring of their postmarketing safety. It is also responsible for providing relief compensation for sufferers from adverse drug reaction and infections by pharmaceuticals or biological products. The PMDA's Office of Biologicals provides consultations concerning clinical trials of new drugs and medical devices and handles biotechnology medicines, including biosimilars [11].

These guidelines are based on the existing processes in Europe and were published by the MHLW in March 2009. Biosimilar drugs are considered as those that are “*equivalent and homogenous to the reference biological product in terms of efficacy, quality and safety.*” This guide also exposes basic requirements necessary to grant the approval of a new biosimilar product to be marketed in Japan. The following requirements must be met [12]:

- It should be established that the manufacturing process for the biosimilar is highly consistent.
- The biosimilar should be fully characterized, using analytical methods.
- The dosage form and route of administration of the biosimilar product should be the same as those of the reference product.
- Long-term, real-time, real-condition stability studies should follow the ICH Q5C guideline (stability testing of biotechnological/biological products).
- A comparison of quality attributes between the biosimilar and the reference product should be conducted.
- Nonclinical studies (including pharmacokinetic, pharmacological, and toxicity studies) that can ensure the safety for administration to humans should be performed and completed prior to initiation of clinical studies.
- Clinical studies are required whenever the data from quality characterization and nonclinical studies is insufficient to demonstrate comparability with the reference product. Clinical studies should be designed based on the data from quality characterization, nonclinical studies, and comparability studies.

The process for approval of a biosimilar in Japan begins when the owner of the drug (legal representative of the laboratory) submits the marketing application, with the requirements listed above, to the MHLW. The application is then passed on to the PMDA, where it follows two necessary but different pipelines. The first is in Conformity Audit Office, which verifies compliance with good clinical and laboratory practices. At this stage, it is confirmed that the information provided by the owner is real. A visit to the premises where the product is manufactured and where clinical studies were conducted is also performed [10]. The second route is through the Office of New Drugs and the Office of Biologics. In these sections, the entire file is reviewed and meetings with the owner are scheduled in order to discuss all aspects of the approval of this product. Once this stage of the review ends, the PMDA produces a detailed report, which is sent to the Pharmaceutical and Food Safety Bureau of the MHLW, which grants or refuses the authorization for marketing the biosimilar, along with its pricing.

In order to obtain the marketing authorization, it is necessary to have had people of Japanese origin included in one of the efficacy and pharmacokinetic studies. If clinical data do not include any people of Japanese ethnicity, it is mandatory to show the similarity between a biosimilar candidate and the reference product according to the International Conference on Harmonization Guidelines [13]. The purpose of this guidance (E5R1) is to facilitate the registration of medicines among ICH regions (European Union, Japan, and the United States) by recommending a framework for evaluating the impact of ethnic factors, upon a medicine effect, its efficacy and safety at a particular dosage, and dose regimen. It provides guidance with respect to regulatory and development strategies that will permit adequate evaluation of the influence of ethnic factors while minimizing duplication of clinical studies and supplying medicines expeditiously to patients for their benefit [14].

While biosimilars cannot be protected by patents, because they have expired, there are regulations to ensure a period of exclusivity in marketing, so that their return is guaranteed. Therefore, research and development should be encouraged by these new types of products. In Japan, this information exclusivity lasts six years, while in the United States it lasts four years and in Europe eight years (Table 3).

It is also noted that, in the case of the United States, there is a distinction between exclusive data and exclusivity in marketing, ensuring a more extensive commercial level to the manufacturer's laboratory biosimilar protection, regardless of any other research and development laboratories conduct to create the original biosimilar. A particular feature of biosimilar approval in the United States is that there are two levels of approval: the biosimilar drug and the biosimilar “Interchangeable Drug” [16].

#### 3.1.3. Biosimilars Approved in Japan

Since the guide for the development of biosimilars in Japan in 2009 was approved, the sale of seven of these types of products has been authorized, the latest of them being insulin glargine BS in January 2015.

Like most pharmaceuticals and biopharmaceuticals, biosimilars require mostly, as shown in Table 4, cooperation or strategic alliances with different laboratories, known as outsourcing firms, for the development and further marketing of a product. Such is the case of the already approved biosimilar “Filgrastim BS3” by Sandoz Laboratory (generic pharmaceuticals division of Novartis), which developed this biosimilar with the collaboration of Sawai Pharmaceutical Co. It is also the case of Nippon Kayaku, which began developing infliximab BS in November 2010, following an agreement with the Celltrion Group (Incheon, South Korea) for the joint development and marketing of this product in Japan [17].

Recently, Lupin Pharmaceuticals, of India, entered into a strategic joint venture with the Japanese-based pharmaceutical company Yoshindo and created a new entity, YL Biologics (YLB). This new company will be responsible for conducting clinical development of certain biosimilars and obtaining marketing authorization in Japan. Its main line of research will be monoclonal antibodies (mAbs) and it expects its first product to be a biosimilar of “Enbrel,” which is marketed in Japan by Takeda Pharmaceutical.

#### 3.1.4. Upcoming Developments in Biosimilars in Japan

One way to analyze the future development of biosimilars is through clinical trials. As of September 2015 [18], 124 worldwide biosimilar clinical trials had been conducted. Of these, only five (representing 4%) were developed in Japan, which shows its remoteness from leading countries, like the United States, with at least 79 clinical studies performed.

According to the public database of the United States [18], at present there are five clinical studies being guided entirely or partly in Japan. It should be mentioned that having a clinical trial assigned to a country does not mean that this study is fully developed in that country, but it may be a partial collaboration in its development. In the case of Japan, we cannot affirm that these trials are wholly developed in that country, as part of them can be carried out in cooperation with third countries.

Below is an outline of the five biosimilars and their area of use of the trials foreclosed to Japan.

(i)* Carboplatin, Paclitaxel, and Gemcitabine Hydrochloride with or without Bevacizumab after Surgery in Treating Patients with Recurrent Ovarian Epithelial Cancer, Primary Peritoneal Cavity Cancer, or Fallopian Tube Cancer*.

 Conditions: clear cell adenocarcinoma; Fallopian tube clear cell adenocarcinoma; Fallopian tube endometrioid adenocarcinoma; Fallopian tube mucinous adenocarcinoma; Fallopian tube serous adenocarcinoma; malignant ovarian mixed epithelial tumor; mucinous adenocarcinoma; ovarian Brenner tumor; ovarian clear cell adenocarcinofibroma; ovarian endometrioid adenocarcinoma; ovarian serous adenocarcinoma; primary peritoneal serous adenocarcinoma; recurrent Fallopian tube carcinoma; recurrent ovarian carcinoma; recurrent primary peritoneal carcinoma; undifferentiated carcinoma; undifferentiated Fallopian tube carcinoma; and undifferentiated ovarian carcinoma.
 Interventions: biological: Bevacizumab (biosimilar); drug: Carboplatin; drug: Docetaxel; drug: Gemcitabine Hydrochloride; other: laboratory biomarker analysis; drug: Paclitaxel; other: quality-of-life assessment.
 Responsibility and sponsor: National Cancer Institute (NCI).
 Listed country: Japan, USA, and South Korea.

(ii)* Carboplatin and Paclitaxel with or without Bevacizumab in Treating Patients with Stage III or Stage IV Ovarian Epithelial, Primary Peritoneal, or Fallopian Tube Cancer*.

 Conditions: Fallopian tube clear cell adenocarcinoma; Fallopian tube endometrioid adenocarcinoma; Fallopian tube mucinous adenocarcinoma; Fallopian tube serous adenocarcinoma; Fallopian tube transitional cell carcinoma; malignant ovarian mixed epithelial tumor; ovarian Brenner tumor; ovarian clear cell adenocarcinoma; ovarian endometrioid adenocarcinoma; ovarian mucinous adenocarcinoma; ovarian serous adenocarcinoma; ovarian transitional cell carcinoma; primary peritoneal serous adenocarcinoma; Stage IIIA Fallopian tube cancer; Stage IIIA ovarian cancer; Stage IIIA primary peritoneal cancer; Stage IIIB Fallopian tube cancer; Stage IIIB ovarian cancer; Stage IIIB primary peritoneal cancer; Stage IIIC Fallopian tube cancer; Stage IIIC ovarian cancer; Stage IIIC primary peritoneal cancer; Stage IV Fallopian tube cancer; Stage IV ovarian cancer; Stage IV primary peritoneal cancer; undifferentiated Fallopian tube carcinoma; and undifferentiated ovarian carcinoma.
 Interventions: biological: Bevacizumab; drug: Carboplatin; other: laboratory biomarker analysis; drug: Paclitaxel; other: Placebo; other: quality-of-life assessment.
 Responsibility and sponsor: National Cancer Institute (NCI).
 Listed country: Japan, United States, Canada, and South Korea.

(iii)* Comparison of CHS-0214 to Enbrel (Etanercept) in Patients with Rheumatoid Arthritis (RA)*.

 Conditions: rheumatoid arthritis.
 Interventions: drug: etanercept; drug: CHS-0214.
 Responsibility and sponsor: Coherus Biosciences, Inc.
 Collaboration: Daiichi Sankyo Co., Ltd.
 Listed country: Japan, United States, Belarus, France, Germany, Hungary, Israel, Poland, Russian Federation, South Africa, Spain, and United Kingdom.

(iv)* GP2013 in the Treatment of Patients with Previously Untreated, Advanced Stage Follicular Lymphoma*.

 Conditions: follicular lymphoma.
 Interventions: biological: GP2013; biological: Rituximab.
 Responsibility and sponsor: Sandoz.
 Collaboration: Novartis Pharmaceuticals.
 Listed country: Japan, Argentina, Australia, Austria, Brazil, Bulgaria, Colombia, France, Germany, Greece, Hungary, India, Ireland, Israel, Italy, Malaysia, Netherlands, Peru, Poland, Portugal, Romania, Russian Federation, South Africa, Spain, Ukraine, and United Kingdom.

(v)* GP2013 in Japanese Patients with CD20 Positive Low Tumor Burden Indolent B-Cell Non-Hodgkin's Lymphoma*.

 Conditions: indolent B-cell non-Hodgkin's lymphoma.
 Intervention: drug: GP2013.
 Responsibility and sponsor: Sandoz.
 Collaboration: Novartis Pharmaceuticals.
 Listed country: Japan.

### 3.2. South Korea

#### 3.2.1. Pharmaceutical and Biopharmaceutical Industry in South Korea

South Korea has been considered as one of the “tiger economies” as a result of the growth experienced by its exports during the second half of the 20th century. There is social health insurance in place since 1977, covering the entire population since 1989, but the country faces a significant challenge due to the aging of the population and the increasing health expenditure [19].

In 2015, the pharmaceutical industry in South Korea became the tenth largest in the world. However, generic production has historically dominated the sector and little attention has been paid to products that are more innovative or require R&D, such as biologicals that need strong investments and involve high risk in their development. This situation has gradually changed over time, through adjustments introduced by successive governments in the country, starting by increasing health coverage in the population and launching institutional initiatives with a high degree of transparency. The aim was to make it attractive for multinational pharmaceutical companies to set up operations in South Korea by allowing good competitive prices, among other things. The public policies implemented in South Korea regarding expenses related to drugs were divided into two different funds. The first one corresponded to a security fund, while the second is related to the Health Care System Reform Act of 2000 or Separation of Prescribing and Dispensing (SPD) of drugs. Furthermore, different pricing policies were established in order to avoid a disproportionate increase in prices to patients caused by the SPD.

The changes made to the intellectual property rights (IPR) law that also contributed to the “modernization” of the pharmaceutical industry in South Korea were due to its improved protection of patent rights for innovative products. In addition, the Free Trade Agreement (FTA) signed with the United States, Europe, and India gave a significant degree of security to the industry, allowing greater foreign investment.

The leading pharmaceutical company in South Korea is Dong-A Pharmaceuticals founded in 1932. In order to grow and reach revenues of nearly US $1 billion, the company established strategic alliances with firms such as Bayer and Meiji, which helped to expand its market and venture into R&D of high-impact biologics. Hence, the company has invested heavily in the development and manufacturing of biosimilars, working with the Incheon Free Economic Zone to build a 142,200 m^2^ plant to manufacture biosimilars in the popular Songdo International Business District of South Korea. The company is currently conducting Phase II clinical trials of DA-6034 (a biosimilar derivative of the original drug eupatilin) for the treatment of dry eyes and also is in the Phase III of DA-3031 (a pegylated human recombinant Granulocyte Colony Stimulating Factor, G-CSF, which is biosimilar to the reference pegfilgrastim, Neulasta) for the treatment of chemotherapy-induced neutropenia in cancer patients. In 2015, Dong-A Pharmaceuticals sold its products to over 40 countries in Europe, Latin America, and Asia [20].

#### 3.2.2. Regulatory Guidelines for Biosimilars in South Korea

The regulatory body for the approval of medicines in South Korea is the Ministry of Food and Drug Safety (MFDS), which is responsible for scientifically evaluating drugs developed by pharmaceutical companies in Korea. The legislation for biosimilars and the guideline for evaluating biosimilars were established by the same agency in July 2009. According to this guideline, a biosimilar in South Korea is defined as a “*biological product that is comparable to an already marketed reference product in terms of quality, safety and efficacy*” [21]. In addition, specific manuals have been developed to assess and facilitate the development of biosimilars for erythropoietin and somatropin (2011), Granulocyte Colony Stimulating Factor (2012), and monoclonal antibody (2013).

The MFDS guideline is harmonized with the World Health Organization guidelines and with the European Union guidelines in its scope and data requirements for authorization. The guideline, therefore, requires a demonstration of similarity and a comprehensive characterization and comparison at the quality level to enable a reduction in the nonclinical and clinical data required for authorization. The regulatory decision is then based on a comprehensive evaluation of quality, safety, and efficacy of data [22].

The authorization process for marketing a biosimilar in South Korea begins with the submission of a dossier by the owner of the product (legal representative of the manufacturer's laboratory) to the MFDS offices. This file must have the following complete documentation [21]:

- Comparability exercise data, including extensive side-by-side characterization, physicochemical properties, biological activity, and immunochemical properties between the biosimilar and the reference product, should be collected.
- Comparative nonclinical studies should be designed to detect significant differences between biosimilar and reference products:
  - in vitro study (receptor binding study and cell proliferation assays);
  - in vivo study (biological/pharmacodynamic studies relevant to the clinical application);
  - toxicity study (at least one comparative repeated dose toxicity study in relevant species, including a toxicokinetic study and antibody measurement).
- Comparative clinical trials are required on the following:
  - pharmacokinetic aspects provide insight into how the drug moves in the body, since it is absorbed, distributed, and biotransformed until eliminated from the body. Moreover, the pharmacodynamics aspects will depend on the pharmacokinetic processes (i.e., pharmacodynamics refers to the effect that makes the drug in the body). Similarly, these pharmacokinetic and pharmacodynamics aspects allow understanding the types of interactions between different drugs, which allow changes in the actions of drugs and their effects on the body;
  - clinical efficacy and safety trials: experimental systematic study is done both in patients and in healthy subjects to evaluate the efficacy and/or safety of one or more therapeutic, diagnostic, or other trials, and to know the effects on the human organism (pharmacodynamics) and/or absorption, distribution, metabolism, and excretion (pharmacokinetics);
  - if applicable, confirmatory pharmacokinetic and pharmacodynamic studies can be used;
  - equivalence design is recommended and equivalence margins should be prespecified and justified;
  - safety data from a sufficient number of patients and study duration should be provided to compare the nature, severity, and frequency of adverse reactions (including immunogenicity study).

For the approval of a biosimilar in South Korea, it is sufficient to demonstrate the similarity in safety and efficacy between the biosimilar and the reference product, in a particular clinical indication. If this is done correctly, the biosimilar authorization for another clinical indication can be obtained. The extrapolation of clinical indications of a biosimilar product is allowed for indications where the postmarketing surveillance period of the reference product has expired and if all of the following conditions are fulfilled [24]:

- Sensitive clinical models to detect potential differences are used.
- Clinical relevant mechanism of action and involved receptor are the same in different indications.
- Safety and immunogenicity have been sufficiently characterized.

In general, the South Korean pharmaceutical companies are stronger in developing generics than in developing more innovative pharmaceutical products. However, in recent years, they have several strategies that have been devised to encourage change. The South Korean government's current investment in the development of biosimilars reached around 35% of the total R&D costs in 2013, while its investment in chemical drug development accounted for just 12%. Another incentive was the creation of a biomedical background of US $80 million in 2009 to encourage the entry of new local companies to the global biopharmaceutical market [25].

#### 3.2.3. Biosimilars Approved in South Korea

Since the guide for biosimilars was published in July 2009, in South Korea, five biosimilars have been approved for marketing; the last one was Brenzys, in September 2015, as Table 5 shows.

Enbrel (etanercept) is the best-selling biological drug in South Korea, with sales exceeding US $4.7 billion in 2014, representing a lucrative market for the development of biosimilars. The patents on Enbrel expire in the United States in November 2028, after Amgen was granted a new patent, while in Europe they expired in August 2015. The approval of Brenzys in South Korea represents the first product approval under Merck's collaboration with Samsung Bioepis, which is a joint venture between the biologics division of South Korean-based multinational Samsung and a United States biotechnology company, Biogen Idec. Samsung Bioepis also announced in January 2015 that it had submitted its etanercept biosimilar candidate, SB4, to the European Medicines Agency (EMA) for approval in the European Union [23].

Celltrion received approval for Remsima in South Korea in July 2012. Further permits followed later on from the European Union, in 2013, and other countries. By obtaining authorization for marketing a biosimilar outside of South Korea, Celltrion became the first laboratory in exporting South Korean biopharmaceutical monoclonal antibodies (mAbs) to international markets, showing a new way to enter the global market for biosimilars [26]. This is confirmed by the company's negotiations to partner with other firms for the development and approval of biosimilars outside Asia. One example of this is its collaboration with Hospira (the world's leading provider of injectable drugs and infusion technologies) to codevelop and commercialize eight biosimilars in Canada, Australia, New Zealand, Europe, and the United States.

#### 3.2.4. Clinical Studies Being Developed in South Korea

As discussed in the previous section, to date (September 2015), 124 clinical trials in biosimilars have been or are being conducted globally, of which six (4.83%) are being carried out in South Korea. The details of the clinical trials carried out in South Korea are presented below [18].

(i)* Carboplatin, Paclitaxel, and Gemcitabine Hydrochloride with or without Bevacizumab after Surgery in Treating Patients with Recurrent Ovarian Epithelial Cancer, Primary Peritoneal Cavity Cancer, or Fallopian Tube Cancer*.

 Conditions: clear cell adenocarcinoma; Fallopian tube clear cell adenocarcinoma; Fallopian tube endometrioid adenocarcinoma; Fallopian tube mucinous adenocarcinoma; Fallopian tube serous adenocarcinoma; malignant ovarian mixed epithelial tumor; mucinous adenocarcinoma; ovarian Brenner tumor; ovarian clear cell adenocarcinofibroma; ovarian endometrioid adenocarcinoma; ovarian serous adenocarcinoma; primary peritoneal serous adenocarcinoma; recurrent Fallopian tube carcinoma; recurrent ovarian carcinoma; recurrent primary peritoneal carcinoma; undifferentiated carcinoma; undifferentiated Fallopian tube carcinoma; and undifferentiated ovarian carcinoma.
 Interventions: biological: Bevacizumab; drug: Carboplatin; drug: Docetaxel; drug: Gemcitabine Hydrochloride; other: laboratory biomarker analysis; drug: Paclitaxel; other: quality-of-life assessment.
 Responsibility and sponsor: National Cancer Institute (NCI).
 Listed country: Japan, USA, and South Korea.

(ii)* Carboplatin and Paclitaxel with or without Bevacizumab in Treating Patients with Stage III or Stage IV Ovarian Epithelial, Primary Peritoneal, or Fallopian Tube Cancer*.

 Conditions: Fallopian tube clear cell adenocarcinoma; Fallopian tube endometrioid adenocarcinoma; Fallopian tube mucinous adenocarcinoma; Fallopian tube serous adenocarcinoma; Fallopian tube transitional cell carcinoma; malignant ovarian mixed epithelial tumor; ovarian Brenner tumor; ovarian clear cell adenocarcinoma; ovarian endometrioid adenocarcinoma; ovarian mucinous adenocarcinoma ovarian serous adenocarcinoma; ovarian transitional cell carcinoma; primary peritoneal serous adenocarcinoma; Stage IIIA Fallopian tube cancer; Stage IIIA ovarian cancer; Stage IIIA primary peritoneal cancer; Stage IIIB Fallopian tube cancer; Stage IIIB ovarian cancer; Stage IIIB primary peritoneal cancer; Stage IIIC Fallopian tube cancer; Stage IIIC ovarian cancer; Stage IIIC primary peritoneal cancer; Stage IV Fallopian tube cancer; Stage IV ovarian cancer; Stage IV primary peritoneal cancer; undifferentiated Fallopian tube carcinoma; and undifferentiated ovarian carcinoma.
 Interventions: biological: Bevacizumab; drug: Carboplatin; other: laboratory biomarker analysis; drug: Paclitaxel; other: Placebo; other: quality-of-life assessment.
 Responsibility and sponsor: National Cancer Institute (NCI).
 Listed country: Japan, United States, Canada, and South Korea.

(iii)* Paclitaxel and Carboplatin with or without Bevacizumab in Treating Patients with Stage II, Stage III, or Stage IV Ovarian Epithelial Cancer, Primary Peritoneal Cancer, or Fallopian Tube Cancer*.

 Conditions: malignant ovarian mixed epithelial tumor; ovarian Brenner tumor; ovarian clear cell cystadenocarcinoma; ovarian endometrioid adenocarcinoma; ovarian mucinous cystadenocarcinoma; ovarian serous cystadenocarcinoma; Stage IIA Fallopian tube cancer; Stage IIA ovarian cancer; Stage IIB Fallopian tube cancer; Stage IIB ovarian cancer; Stage IIC Fallopian tube cancer; Stage IIC ovarian cancer; Stage IIIA Fallopian tube cancer; Stage IIIA ovarian cancer; Stage IIIA primary peritoneal cancer; Stage IIIB Fallopian tube cancer; Stage IIIB ovarian cancer; Stage IIIB primary peritoneal cancer; Stage IIIC Fallopian tube cancer; Stage IIIC ovarian cancer; Stage IIIC primary peritoneal cancer; Stage IV Fallopian tube cancer; Stage IV ovarian cancer; Stage IV primary peritoneal cancer; and undifferentiated ovarian carcinoma.
 Interventions: biological: Bevacizumab; drug: Carboplatin; procedure: computed tomography; drug: Paclitaxel; procedure: therapeutic conventional surgery.
 Responsibility and sponsor: National Cancer Institute (NCI).
 Listed country: United States, Canada, and South Korea.

(iv)* Radiation Therapy with or without Trastuzumab in Treating Women with Ductal Carcinoma In Situ Who Have Undergone Lumpectomy*.

 Conditions: ductal breast carcinoma in situ; HER2/Neu positive.
 Interventions: other: laboratory biomarker analysis; biological: trastuzumab; radiation: whole breast irradiation.
 Responsibility and sponsor: National Cancer Institute (NCI).
 Listed country: United States, Canada, South Korea, and Puerto Rico.

(v)* An Extension Study to Demonstrate the Equivalence of Long-Term Efficacy and Safety of CT-P13 in Patients with Ankylosing Spondylitis Who Were Treated with Infliximab (Remicade or CT-P13) in Study CT-P13 1.1*.

 Conditions: ankylosing spondylitis.
 Interventions: biological: infliximab.
 Responsibility and sponsor: Celltrion.
 Listed country: South Korea.

(vi)* Demonstrating the Equivalence of CT-P10 to MabThera with respect to the Pharmacokinetic Profile in Patients with Rheumatoid Arthritis*.

 Conditions: rheumatoid arthritis.
 Interventions: biological: Rituximab.
 Responsibility and sponsor: Celltrion.
 Listed country: South Korea.

### 3.3. Malaysia

#### 3.3.1. Pharmaceutical and Biopharmaceutical Industry in Malaysia

The demand for quality health has been considered crucial by the Malaysian government and its population, and living conditions in this country are improving on a yearly basis. The Malaysian health system has evolved greatly in recent decades and has been listed by the World Health Organization (WHO) as having a universal healthcare system. Malaysia currently invests 7.25% of the Gross Domestic Product (GDP) of the country in health.

The pharmaceutical industry is one of the sectors that the Malaysian government has prioritized for its promotion and development. Pharmaceuticals in this country are divided into four broad categories:

1. prescription medicines,
2. over-the-counter (OTC) products,
3. traditional medicines,
4. health/food supplements.

An update by the Malaysian Investment Development Authority (MIDA) [27] shows that pharmaceutical companies are mainly small- and medium-sized firms engaged in the production of generic drugs, traditional medicines, and herbal supplements, as well as in contract manufacturing for foreign multinational corporations (MNCs).

Among the major local companies are Pharmaniaga Manufacturing Berhad, Hovid Berhad, CCM Pharmaceuticals Division (comprises CCM Duopharma Biotech Berhad, CCM Pharmaceuticals Sdn Bhd, and Innovax Sdn Bhd), and Kotra Pharma (M) Sdn Bhd. Some of the foreign-owned companies with manufacturing presence in the country include Y.S.P. Industries (M) Sdn Bhd (Taiwan), Sterling Drug (M) Sdn Bhd (the manufacturing arm of GlaxoSmithKline from the United Kingdom), Ranbaxy (M) Sdn Bhd (India), Xepa-Soul Pattinson (M) Sdn Bhd (Singapore), and SM Pharmaceuticals Sdn Bhd (India).

The large MNCs—such as Pfizer, Schering Plough, Novartis, Eli Lilly, and AstraZeneca—are mainly licensed importers. Their products, mostly branded drugs, are distributed by locally incorporated companies.

Biosimilars are expected to have a comparable, if not greater, impact on the biopharmaceutical industry than that of generics. Malaysia offers a more competitive cost option to investors due to its enabling environment. A large number of first-generation biopharmaceutical products are nearing maturity and major biopharmaceutical companies are likely to move these out to countries that offer a good value preposition, like Malaysia [27].

Currently, in Malaysia, local and foreign players are already engaged in activities like the production of active pharmaceutical ingredients (APIs) and applications for good manufacturing practice (cGMP) compliant services at the United States Food and Drug Administration (FDA) and Europe, the Middle East and Africa (EMEA) focusing on monoclonal antibodies and recombinant proteins. Malaysia has also developed specialized parks to cater to the needs of specific industries, which are technology-intensive and research-intensive. Examples of these parks are the Technology Park Malaysia in Bukit Jalil, Kuala Lumpur, and the Kulim Hi-Tech Park in the northern state of Kedah. These parks comprise state-of-the-art buildings with specific functions and a fully integrated high-technology park. Other specialized parks developed by the Malaysian government agencies are the following: Bio-XCell Malaysia, Penang Science Park, Kulim Hi-Tech Park (KHTP), and techpark@enstek [26].

Malaysia has been concerned with developing a powerful system of intellectual property protection, in accordance with international standards, akin to protect inventions and foreign and local investors.

Malaysia is a party to the following treaties:

- World Intellectual Property Organization (WIPO), 1967;
- Paris Convention for the Protection of Industrial Property, 1883;
- Berne Convention for the Protection of Literary and Artistic Works, 1886;
- Trade-Related Aspects of Intellectual Property Rights (TRIPS) Agreement, 1994.

#### 3.3.2. Regulatory Guidelines for Biosimilars in Malaysia

The Ministry of Health of Malaysia, through the National Pharmaceutical Control Bureau (NPCB), is the national authority that assures the quality of medicines in the country. The NPCB is responsible for ensuring that all pharmaceutical and biopharmaceutical products developed and commercialized in the country meet the standards of quality, safety, and efficacy as required by law [29].

Unlike other countries that have been studied in this work, there is no elaboration of an exclusive guideline for biosimilars, only an addition to the law previously created for biological drugs (Drug Registration Guidance Document (DRGD)), as a subcategory of biological products. This implies that in order to obtain approval for a biosimilar it must meet all of the quality, safety, and efficiency requirements for any product of biological origin. No distinction is made between a new biological product and the one that has been marketed for many years and is now based in the development of a biosimilar.

The requirements for the registration of biologics/biopharmaceuticals are guided by the ASEAN Common Technical Dossier (ACTD) and the Drug Registration Guidance Document (DRGD). The requirements are

- administrative information;
- product quality data (clinical analysis);
- product safety data (nonclinical analysis);
- clinical data, demonstrating clinical efficacy and capacity to meet therapeutic claims, through clinical studies.

The process for registering a biosimilar begins with an online submission to the NPCB by the legal representative of the new product. With this, the office generates an appointment for the representative to present all of the information necessary to meet the approval requirements set forth, such as analytical methods and quality validation documentation. With this information, the NPCB consults with experts, usually external, such as academics, clinical advisors, and health and biological experts. Together, they produce an evaluation report that is sent to the Drug Control Authority (DCA) of NPCB, which defines whether or not the biological product can be approved to be marketed in Malaysia [29].

#### 3.3.3. Biosimilars Approved in Malaysia

Since the biosimilar guideline was published in July 2008, in Malaysia, five biosimilars have been approved for marketing. The latest was Insugen, in January 2014, as Table 6 shows.

All biosimilars that have been approved so far are of a relatively small size and reduced complexity. But they must not be confused with generic molecules that are even smaller and simpler. While they all originate from a reference, biological products have been marketed in Malaysia for several years and with higher sales volumes. It is also striking that none of them is developed by local biopharmaceutical companies or at least with their collaboration.

Nevertheless, apparently, this would be changing as Viropro, Inc., and Oncobiologics, Inc., signed a collaboration agreement for the development of biosimilars. Under this agreement, Viropro will have the rights to manufacture six monoclonal antibody products being developed by Oncobiologics for commercialization in more than 70 emerging market countries (excluding China). Viropro will have exclusive commercialization rights to the six biosimilars for Malaysia. In addition, the companies will comanage Viropro's Penang, Malaysia, Alpha Biologics biomanufacturing subsidiary. The six biosimilars are versions of Humira, Rituxan, Avastin, Herceptin, Erbitux, and one other nondisclosed biotherapeutic [30].

#### 3.3.4. Developing Clinical Trials in Malaysia

Of the 124 clinical trials being carried out in biosimilars in the world, only two of them (1.6%) are being conducted in Malaysia [18]. Analyzing the information in more detail, we note that, of the trials currently underway in Malaysia, none of them involved a local biopharmaceutical company, indicating the lack of commitment or interest of these companies in the development of biosimilars. The details of the clinical studies carried out in Malaysia are presented below.

(i)* Study of RTXM83 plus CHOP Chemotherapy versus a Rituximab plus CHOP Therapy in Patients with Non-Hodgkin's Lymphoma*.

 Conditions: diffuse large B-cell lymphoma.
 Interventions: biological: RTXM83.
 Responsibility and sponsor: mAbxience SA.
 Collaborations: Pisa Farmacética; Laboratorios de Productos Éticos CEISA; Laboratorio Elea SACIF y A; Tecnoquímicas; Innogene Kalbiotech Pte. Ltd.; and Libbs Farmacêutica Ltda.
 Listed country: Malaysia, Argentina, Brazil, Colombia, India, Indonesia, Mexico, Paraguay, Philippines, South Africa, and Thailand.

(ii)* GP2013 in the Treatment of Patients with Previously Untreated, Advanced Stage Follicular Lymphoma*.

 Conditions: follicular lymphoma.
 Interventions: biological: GP2013; biological: Rituximab.
 Responsibility and sponsor: Sandoz.
 Collaborations: Novartis Pharmaceuticals.
 Listed country: Malaysia, Argentina, Australia, Austria, Brazil, Bulgaria, Colombia, France, Germany, Greece, Hungary, India, Ireland, Israel, Italy, Japan, Netherlands, Peru, Poland, Portugal, Romania, Russian Federation, South Africa, Spain, Ukraine, and United Kingdom.

## 4. Conclusion

Much of the biopharmaceutical industry is betting towards a future in biosimilars, giving a second life to biological molecules that are reaching the end of their period of intellectual property protection and, at the same time, a means to increase their profitability in the medium term, which new biological molecules tend to lack. The production and marketing of biosimilars involve lower risks and costs. In addition, it is considered that the biosimilar industry in 2015 covers globally only 1% of the total production of biological molecules because patents have only recently begun to expire, so that a promising future is announced for the next decade.

The East and Southeast Asian countries discussed in this paper are highly developed in relation to the institutional and technological environment, although, interestingly, they show some delay in the effective implementation of clinical trials in their own countries and, consequently, in the development of biosimilars. This is reflected in their participation in various clinical trials of biosimilars around the world, which are actually only 124. Of these, Japan is involved in only five and contributes to 4%, while South Korea owns six clinical trials, with a contribution of 4.83% and, finally, Malaysia falls behind with only two trials and 1.6% of the total contribution. This does not mean that companies in these countries do not develop clinical trials in other countries or in their own research departments. There is evidence that the South Korean company Celltrion, for example, develops up to eight clinical trials in other countries. Perhaps the associated costs and regulations involve a major setback and make them more profitable in countries with less regulatory restrictions, as in the case of emerging countries.

There is a cost advantage for the producer of a biosimilar, but the original owner of the biological molecule should not be forgotten, since the company continues to produce and market its product and should have a competitive advantage and comparative processes and marketing channels, taking advantage of this “inertia” provided by the production and marketing of the product for over 20 years. However, we would expect a strategic shift of these firms, perhaps through a reduction in their prices in order to remain competitive.

Biosimilars are excellent news, not only for biopharmaceuticals, but also for patients due to the reduction of costs and prices up to 36% in comparison to the original drug. The great boom of biosimilars is to come in the next few decades, but there is still a need to unify criteria and regulations globally in order for their success to be full and widespread.

## Figures and Tables

**Table 1:** Differences between chemical, biological, generic, and biosimilar drugs.

Parameter	Pharmaceutical industry		Biopharmaceutical industry	Biosimilar industry
	Chemical drug	Generic drug	Biological drug	Biosimilar drug
Synthesis	Production of original chemical formula	Copy from the original chemical formula	Since the insertion of a gene that plays a cell clone molecule	Development derived from the original biological molecule
Size	100–1000 Da	100–1000 Da	10.000–300.000 Da	10.000–300.000 Da
Glycosylation process	Zero	Zero	Several	Several
Molecular structure	Simple	Simple	Complex	Complex
Ability to generate immunity	Low	Low	Medium-high	Medium-high
Drug development time	7–10 years	1–3 years	10–15 years	6–9 years
Costs	US $500–800 M	<US $1-2 M	US $800–1300 M	US $10–250 M
Characterization analysis laboratory	N/A	There are techniques to identify similarity to the original drug	N/A	No identification technique equality of the molecule Clinical studies are needed

Source: adapted from Zelenetz et al. [5].

**Table 2:** Leading biopharmaceuticals sold in Japan, 2015.

Product name	Active agent	Company selling	Company origin
Remicade	Antirheumatic drug	Mitsubishi Tanabe Pharma	J&J
Avastin	Anticancer agent	Chugai Pharmaceutical Co.	Loches
NESP	Renal anemia	Kyowa Hakko Kirin	Kyowa Hakko Kirin
Enbrel	Antirheumatic drug	Takeda	Amgen, Inc.
Herceptin	Anticancer agent	Chugai Pharmaceutical Co.	Loches
Lucentis	Wet age-related macular degeneration	Novartis AG	Roche/Novartis
Rituxan	Anticancer agent	Chugai Pharmaceutical Co.	Roche/Novartis
Humira	Antirheumatic drug	Eisai Co., Ltd.	Abbott
Mirusera	Renal anemia	Chugai Pharmaceutical Co.	Chugai Pharmaceutical Co.

Source: Mizuho Bank Industry Research, 2014 [7].

**Table 3:** Data on exclusivity following approval of biosimilars.

Country	Data on exclusivity period
Japan	Eight years of data exclusivity
Canada	Eight years of data exclusivity
United States	Four years of data exclusivity/eight years of market exclusivity
Europe	Ten years of data exclusivity
South Korea	Eight years of data exclusivity
Singapore	Five years of data exclusivity
Malaysia	Five years of data exclusivity
Australia	Five years of data exclusivity

Source: RAPS, 2015 [15].

**Table 4:** PMDA approved biosimilars in Japan.

Product name	Therapeutic area	Authorization date	Manufacturer/company name
Somatropin BS	Growth hormone deficiency Turner syndrome	Jun. 22, 2009	Sandoz
Epoetin alfa BS	Anemia/renal anemia	Jan. 20, 2010	JCR Pharmaceuticals
Filgrastim BS 1	Cancer/neutropenia/hematopoietic stem cell transplantation	Nov. 21, 2012	Fuji Pharma Co., Mochida Pharmaceutical
Filgrastim BS 2	Cancer/neutropenia/hematopoietic stem cell transplantation	Feb. 28, 2013	Teva Pharma Japan, Nippon Kayaku Co.
Filgrastim BS 3	Cancer/neutropenia/hematopoietic stem cell transplantation	Mar. 24, 2014	Sandoz Österreich
Infliximab BS	Crohn's disease/rheumatoid arthritis/ulcerative colitis	Jul. 4, 2014	Nippon Kayaku Co.
Insulin glargine BS	Growth hormone deficiency/Turner syndrome	Jan. 19, 2015	Elli Lily/Boehringer Ingelheim

Source: Generics and Biosimilars Initiative, 2016 [17].

**Table 5:** MFDS approved biosimilars in South Korea.

Product name	Active substance	Therapeutic area	Authorization date	Manufacturer/company name
Remsima	Infliximab	Ankylosing spondylitis Crohn's disease Psoriasis/rheumatoid arthritis/ulcerative colitis	Jul. 23, 2012	Celltrion
Omnitrope	Somatropin	Pituitary dwarfism, Prader-Willi syndrome, and Turner syndrome	Jan. 2014	Sandoz
Herzuma	Trastuzumab	HER_2_ + breast cancer Advanced (metastatic) stomach cancer	Jan. 15, 2014	Celltrion
Davictrel	Etanercept	Ankylosing spondylitis Psoriasis/rheumatoid arthritis/psoriatic arthritis	Nov. 11, 2014	Hanwha Chemical
Brenzys	Etanercept	Ankylosing spondylitis Psoriasis/rheumatoid arthritis/psoriatic arthritis	Sep. 3, 2015	Merck/Samsung Bioepis

Source: MFDS, 2015 [23].

**Table 6:** DCA approved biosimilars in Malaysia.

Product name	Active substance	Therapeutic area	Authorization date	Manufacturer/company name
SciTropin	Somatropin	Growth disturbance in children and growth hormone deficiency in adults	Aug. 2010	Sandoz
Binocrit	Epoetin alfa	Renal anemia, cancer, and predonation preparation for autologous and allogeneic blood transfusion	Mar. 2011	Sandoz
Zarzio	Filgrastim	Cancer, HSCT, and chronic neutropenia	Mar. 2012	Sandoz
Nivestim	Filgrastim	Cancer, HSCT, and chronic neutropenia	Aug. 2013	Hospira
Insugen	Recombinant human insulin	Diabetes mellitus	Jan. 2014	Biocon

Source: DCA, 2014 [28].
